# Supplementary material for: Elderly care expectation how to influence the fertility desire: Evidence from Chinese general social survey data
Source: PLoS One. 2025 May 7;20(5):e0318628. doi: 10.1371/journal.pone.0318628 (PMC12057963; doi:10.1371/journal.pone.0318628)
Supplement: Attachment — Submitted filename: Response to Reviewers.docx [file pone.0318628.s002.docx]

Dear Reviewers,

Thank you very much for your thorough review and valuable feedback on my manuscript.

**First**, I greatly appreciate your comment regarding the appropriateness and rigor of my statistical analysis.In this study, I employed logistic regression model, linear regression model and the Propensity Score Matching model, which are widely recognized and recommended in the field of social statistics for analyzing data of this nature. I selected these methods because they are well-suited to handle our type of data and effectively address our research questions. Specifically, "Whether to give birth" is a binary variable, so it is suitable for logistic regression model analysis. For the “expected number of births” and “gender preference”, they are more suitable for the least squares regression model to analyze.The non-experimental data used in the study may have data bias and the influence of confounding variables is more, so the propensity score matching model can be used to further verify the analysis.The role of PSM is to reduce confounding bias(reduces the impact of these confounding variables by balancing the distribution of covariates between groups, so as to estimate the treatment effect more accurately), simulate randomized trials(simulates the conditions of randomization experiments by matching individuals with similar propensity scores,thereby approximating the causal effect), and improve inter-group comparability(it ensures that the two groups are comparable in baseline characteristics by matching individuals with similar characteristics in the treatment group and the control group, thereby improving the reliability of the results).

To ensure the rigor of our analysis, I conducted several steps during the data preprocessing phase, including outlier handling, missing data imputation etc, and adhered strictly to relevant statistical guidelines and protocols.The results have shown consistency across multiple repeated experiments, further validating the reliability of our methods.

**Second**,I greatly appreciate your comment regarding the need for a more structured abstract. I agree that a well-organized abstract can significantly improve the clarity and accessibility of the article.In response to your suggestion, I have revised the abstract to follow a structured format,including the following sections:Background,Methods,Results, and Conclusions. This new structure provides a clearer and more comprehensive overview of our study, making it easier for readers to understand the key aspects of our research.

Below is the revised abstract for your review:

///Background

The changes of population structure will have profound impact on economic and social development. In recent years, the continuous intensification of population aging and the persistent decline of fertility rates have posed a certain challenge to the stability of population structure in China.This paper combines the two social hot issues of elderly care and fertility, and puts forward relevant policies and enlightenment through quantitative analysis and research,so as to promote better social development.

Methord

Based on the data of the Chinese General Social Survey (CGSS) in 2017 and 2021, this paper uses the logistic regression model, linear regression model and the Propensity Score Matching model to analyze the influence of the elderly care expectation on residents' fertility desire. The elderly care expectations include government elderly care, self-care, offspring elderly care and responsibility sharing.The fertility desire includes ‘whether to give birth’, ‘quantity’,‘gender preference’ and ‘fertility echelon’.

Result

The research resules shows that: first, government elderly care expectations not only significantly reduces residents' willingness to have children, but also has an inhibitory effect on lower fertility echelons, but has a promoting effect on higher fertility echelons. Second, the self-care expectations significantly reduces residents' willingness to have children and has a significant negative impact on their expected fertility quantity, especially has a significant inhibitory effect on lower fertility echelons.Third, offspring elderly care expectations significantly increases residents' willingness to have children and has a significant positive impact on their expected fertility quantity, especially for increasing residents' willingness to have a second child, and residents with offspring elderly care expectations have a higher preference for boys.

Conclusion

This study highlights the complex relationship between elderly care expectation and fertility desire, offering valuable insights for policymakers. The concept of fertility echelons provides a nuanced understanding of the dynamic impacts on fertility preferences. By addressing both elderly care and fertility challenges, this research contributes to the development of strategies for sustainable social progress.The concept of fertility echelon provides a nuanced understanding of the dynamic impacts on fertility preferences. By addressing both elderly care and fertility challenges, this research contributes to the development of strategies for sustainable social progress.///

I believe that these changes address your concern and enhance the overall quality of the article.

**Third**,You mention the format of the paper and the long citation. I have modified some of the formats and appropriately reduced the content of the citation to meet academic standards.

**Fourth**,you also mention the references, the adaptability of the research results and the limitations of the research.We acknowledge that the initial version lacked sufficient references to studies from other countries.Although it is confirmed that the expectation of elderly care has a significant impact on fertility desire, it is limited to China. As we all know, the population situation and cultural background of China are relatively special, and the Chinese people have the traditional ideas of ' raise children to provide against old age ' and ' carry on the family line ',so the research results may not be applicable to other countries.In this study, I added the latest international research literature related to this study, and the results found that the increase in caregiving responsibilities toward elderly parents will lead to an immediate and substantial decline in the fertility expectations of adult children, which are similar to our study.So it can show that the results of this study have certain applicability and provide a broader context for our research.

**Fifth**,about the problem of ‘lacks clarity’, I have been modified in the article.I have added appropriate annotations below the table and added appropriate interpretation of psm in the article to facilitate the reader's understanding, including the interpretation of ' Treated ', ' Controls ' and ' Difference ' in the table, and the interpretation of C and K in psm. After all the matching, more than 95 % of the samples are matched, indicating that the matching effect is good. However, due to the existence of different elderly care expectations and the existence of three outcome variables, the number of matching is more, so the effect of each sample matching is not described in detail in the article.

**Sixth**,We acknowledge that the original manuscript was somewhat cumbersome to read due to excessive use of abbreviations. In the revised version,we have tried to simplify the language and reduced unnecessary descriptive content to improve readability.

**Seventh**,We have expanded the discussion of the statistical results to address your concerns:explained why the consistency between the PSM results and logistic regression results supports hypotheses H1a, H1b, and H1c . The results of logistic regression show that expectations of offspring caring for parents have a significant positive impact on residents' desire to have children, while personal and governmental elderly care expectations have a significant negative influence. According to the results in Table 7, when cp, op and gp were selected, the values of difference were 0.024, -0.081 and-0.060, respectively, indicating that the average fertility intention of the treatment group was higher than that of the control group when cp was selected. When op was selected, the average fertility intention of the treatment group was lower than that of the control group. When gp was selected, the average fertility intention of the treatment group was lower than that of the control group.The results of the two studies were the same, which confirm H1a,H1b,H1c.

**Eighth**,i want to clarify the significance of the large Z-values in Table 8 here.The greater the z value, the stronger the significance of the difference between the treated group and the control group.The z value of cp is greater than that of op and gp, indicating that when cp is selected, the difference between the treated group and the control group is more significant, which is the same as the regression results in Table 4, indicating that the results are reasonable.

I am more than willing to provide additional details or make necessary revisions. If you could point out specific areas of concern, i would be able to address them more precisely.Once again, we sincerely thank you for your valuable feedback, which has significantly improved the quality of our manuscript. We hope that the revised version meets your expectations and the standards of the journal.

Best regards,

Yang Jia
